# The identification of effective welfare indicators for laboratory-housed macaques using a Delphi consultation process

**DOI:** 10.1038/s41598-020-77437-9

**Published:** 2020-11-23

**Authors:** Melissa A. Truelove, Jessica E. Martin, Fritha M. Langford, Matthew C. Leach

**Affiliations:** 1 Yerkes National Primate Research Center, Emory University, 954 Gatewood Rd NE, Atlanta, GA 30329 USA; 2 Royal (Dick) School of Veterinary Studies and The Roslin Institute, University of Edinburgh, Edinburgh, EH25 9RG UK; 3 Animal and Veterinary Sciences, SRUC, West Mains Road, Edinburgh, EH9 3JG UK; 4 School of Natural and Environmental Sciences, Newcastle University, Newcastle upon Tyne, NE1 7RU UK

**Keywords:** Animal behaviour, Experimental models of disease

## Abstract

Despite the importance for both animal welfare and scientific integrity of effective welfare assessment in non-human primates, there has been little or no consensus as what should be assessed. A Delphi consultation process was undertaken to identify the animal- and environment-based measures of welfare for laboratory-housed macaques and to determine their relative importance in on-site welfare assessments. One-hundred fifteen potential indictors were identified through a comprehensive literature search, followed by a two-round iterative electronic survey process to collect expert opinion. Stable group response and consensus about the validity, reliability, and feasibility of the proposed indicators (67.5% agreement) was achieved by the completion of Round Two. A substantially higher proportion of environment-based measures (72%: n = 44/61) were considered as valid, reliable, and feasible compared to the animal-based measures (22%: n = 12/54). The indicators that ranked most highly for assessing welfare were the presence of self-harm behaviours and the provision of social enrichment. This study provides an empirical basis upon which these indicators can be validated and then integrated into assessment tools developed for macaques and emphasises the need to include both animal- and environment-based indicators for accurate welfare monitoring.

## Introduction

The effective assessment of macaque welfare is critical for determining the current welfare state of animals, maintaining and then improving this state, and determining the effectiveness of any efforts made to improve their welfare. Globally, macaques are the most commonly used non-human primate (NHP) in research^1,2^, for example 3000 procedures carried out on them in United Kingdom^3^ and comprising 75% of the NHPs expected to be used in research in the United States in 2019^4^. Although NHP research forms a small proportion of the research carried out on animals (e.g. < 0.5% in the United States^5^), primates play a critical role in some of the most important biomedical research undertaken^6^. The welfare of these animals is of increasing focus for the public, those who care for these animals, those who regulate their use, and those who use them in their research. The established driver for this is our appreciation for their capacity to suffer and experience positive welfare^7^, which appears to be potentially greater than most other laboratory animal species. Added to this, is the increasing understanding of the negative impact of poor welfare on the validity of the data collected from such animals^8^. The more intact the animal, the better the research model^9,10^. For example, provision of species-appropriate environmental enrichment, like access to social partners, enhances NHP welfare, and consequently, experimental validity and reproducibility^11,12^.

Currently, there is no consensus on the welfare indicators for laboratory-housed macaques beyond those that are often used as evaluative tools to determine compliance with minimum standards of care which are more akin to risk assessments. These are primarily legislative or accreditation-driven, and so are often more focused on ensuring intact animal models for quality science^13^. They tend to rely heavily on the assessment of environmental parameters describing management practices and environment features (e.g. inputs), as they are objective and easy to measure accurately^14,15^. However, to more effectively evaluate the welfare state of an individual per se, we should be assessing quantifiable animal-based outcome indicators, as they represent animals’ reaction (e.g. behaviour, physiology, and health) to the environment (outputs)^14,16–19^ and other aspects of captivity. The use of animal-based output measures either alone^20^ or in combination with the assessment of environment-based inputs^21,22^ is critical for robust and effective assessment of welfare assessment that reflects welfare as a dynamic entity^23,24^ on a multi-dimensional continuum rather than as “good” or “poor”^17,25,26^. The modern assessment of farm animal welfare has been using such an approach for more than 40 years (e.g. Welfare Quality assessment protocols^27^) and was suggested as a framework for how to evaluate and promote captive NHP welfare 30 years ago^28^.

To effectively evaluate welfare of laboratory-housed macaques and, in turn, make recommendations for future improvement requires effective assessment of welfare, as these allow us to benchmark the current welfare state and identify any improvement. Such benchmarking ensures the existing empirically based refinement recommendations involving human influence, the environment, and management practices^29–31^ that are often underutilized (e.g. positive reinforcement training^32–34^) are effectively applied, and new means of defining and mitigating poor welfare in captive NHPs (e.g. self-injurious behaviour^35,36^, output of the hypothalamic–pituitary–adrenocortical axis^37^) are tested and validated. Development of an additional welfare tool for use in regulatory assessment, for in-house benchmarking, and to increase public transparency and trust, would be beneficial to ensure welfare standards are met and to strengthen scientific validity of primate-based studies.

Despite some efforts to identify effective indicators for assessing macaque welfare^38,39^, no attempt has been made to collectively define what they are, whether they are effective (i.e. valid, reliable and feasible), and how they can be used. Parameters should show content validity (i.e. assessing all aspects of welfare), reliability (i.e. can be consistently measured across and between users), and feasibility (i.e. can be measured with limited time, resources, and within facility constraints)^14,40–43^. Validation involves initially establishing that an indicator meets each of these criteria in a captive environment (see Welfare Quality project as a model^44^). This process is necessary to design and implement assessment tools for the identification of both the causes of and areas for improvement for diminished welfare^41^. One method of identifying indices thought to reflect an animal’s welfare is the Delphi consultation, which consists of a multi-round process (usually two to three) of questionnaire administration and controlled feedback to a panel of experts (i.e. stakeholders who interact with NHPs in varying capacities) from various backgrounds who participate anonymously; their responses are used to reach a group consensus on a topic, as indicated by response stability between rounds^45–48^. This approach has been used previously to identify welfare indicators for species maintained on farm (e.g. dairy cows, laying hens, pigs^49^; broiler chickens^50^), in semi-captive environments (e.g. elephants involved in wildlife tourism^51^; dogs in catch-neuter-release programs^52^), and in the laboratory (mice^21,22^). This systematic approach is more rigorous than other group consensus approaches, like case studies or focus groups, because of its reliance on scientific evidence and involvement of expert opinion, which results in enhanced decision-making, identification of quality indicators, and the confluence of expertise^47^.

The aim of this study was to use a Delphi consultation process to identify and determine the relative value of different potential measures of laboratory-housed macaque welfare based on the validity, reliability, and feasibility of the measures.

## Results

### Demographics

Of the 39 experts who completed the two survey rounds, 74% had some form of post-graduate qualification (n = 29/39), and 62% had more than 10 years’ experience with macaques (n = 24/39). Eighty percent of the experts (n = 31/39) had experience with both of the two macaque species most utilized in research, *M. mulatta* (n = 39/39; 100%) and *M. fascicularis* (n = 31/39; 80%). Ninety percent of the experts were North American (n = 35/39).

Behavioural management, animal welfare, and research-oriented experts comprised 74% of the panel (n = 29/39), with veterinary medicine (18%; n = 7/39), and animal care, colony management, and unlisted but related occupations (8%; n = 3/39) making up the remainder of the panel. (see Supplementary Tables S1–S3 online for all demographics data).

### Survey

#### Consensus and group stability

There was a significant effect of individual respondent (F_1, 26906_ = 4.71, p = 0.030), survey round (F_1, 26906_ = 10.22, p = 0.001), and indicator (F_1, 26906_ = 286.54, p < 0.001) on survey response (Table 1). Strong stability in individual responses across both within and between rounds is illustrated by back-transformed means (Table 1), which approached a high degree of stability in Rounds One and Two and did not appreciably change between these rounds.

**Table 1:** Consensus and group stability of welfare indicators: generalized linear mixed model (GLMM) 1 [respondent, round, indicator (fixed effects); respondent (random effect)].

	Mean	Back-transformed mean	SEM (avg)	SEM (lower)	SEM (upper)
**Round**					
1	0.8349	0.6974	0.01908	0.01891	0.01925
2	0.9211	0.7153			

Group stability, or the consistency of participant responses between successive iterations of a survey^53^, amongst the 39 experts who participated in both rounds was assessed with Krippendorff’s alpha test of the responses they provided on 115 indicators with three response types (validity, reliability, feasibility). The group’s level of disagreement across all 345 items was high in both rounds (Round 1, α = 0.1947; Round 2, α = 0.1358); however, levels remained relatively consistent between rounds (Δ 0.0589) and the movement that did occur was in the direction of agreement (signifying convergence, i.e. consensus).

Across the 115 proposed welfare indicators, the overall consensus (for validity, reliability, feasibility) was 67.5% (n = 233/345) agreement. Within this, consensus for validity, reliability, and feasibility was 73% (n = 84/115), 63% (n = 72/115), and 67% (n = 77/115) respectively. This varied according to indicator type, with 63% respondent agreement for animal-based indicators and 86% for environment-based indicators.

Fifty-six of the 115 indicators (49%) were considered valid, reliable, and feasible at the set level of ≥ 70% agreement. This comprised of 12 animal- and 44 environment-based measures (Tables 2, 3). Consensus that an indicator was less valid, reliable, or feasible was reached for two indicators: acute phase proteins and telomere length (animal-based measures). The remaining indicators either approached consensus (65–69.99%) for either validity, reliability, or feasibility, or there was mixed agreement amongst the experts (dissensus). Supplementary Table S4 online shows a complete listing of agreement for the 115 welfare indicators by response type.

**Table 2:** Animal-based welfare indicators reaching consensus by percentage agreement scores.

Indicator description	Valid (%)	Reliable (%)	Feasible (%)	Composite score (avg of V + R + F) (%)
Appetite	92.3	79.5	82.1	84.6
Blood in waste	94.9	89.7	82.1	88.9
Body weight	79.5	79.5	94.9	84.6
Discharge	87.1	82.1	82.1	83.8
Dyspnoea	94.9	82.1	89.7	88.9
Huddled posture	89.7	76.9	92.3	86.3
Injuries, environmental	84.6	71.8	82.1	79.5
Injuries, non-human primate	92.3	79.5	82.1	84.6
Mortality	79.5	82.1	79.5	80.3
Prolapse	71.8	71.8	76.9	73.5
Self-harm behaviours	100.0	87.2	94.9	94.0
Stereotypical behaviours	82.1	87.2	97.4	88.9

**Table 3:** Environment-based welfare indicators reaching consensus by percentage agreement scores.

Indicator description	Valid (%)	Reliable (%)	Feasible (%)	Composite score (%)
Animal caregiver observations	97.1	76.5	91.2	88.3
Behavioural management program	100	88.2	94.1	94.1
Browse provision	88.2	76.5	91.2	85.3
Cage complexity	88.2	79.4	73.5	80.4
Cage dimension	79.4	85.3	88.2	84.3
Cage furniture	97.1	91.2	88.2	92.2
Cage position	85.3	79.4	88.2	84.3
Chair restraint frequency	85.3	76.5	79.4	80.4
Destructible enrichment	94.1	82.4	88.2	88.2
Disease surveillance	100	85.3	91.2	92.2
Experiments, lifetime	73.5	67.6^^^	85.3	79.4
Field of view	85.3	82.4	76.5	81.4
Food enrichment	97.1	94.1	97.1	96.1
Food variety	88.2	82.4	97.1	89.2
Health monitoring	100	94.1	100	98.0
Hear other NHPs	88.2	91.2	97.1	92.2
Humane euthanasia program	100	94.2	100	98.1
Humidity	76.5	85.3	88.2	83.3
Inoculations, lifetime	79.4	82.4	88.2	83.3
Light intensity	79.4	85.3	88.2	84.3
Manipulanda	88.2	79.4	94.1	87.2
Moves, lifetime	91.2	76.5	73.5	80.4
Novelty exposure, intentional	85.3	82.4	97.1	88.3
Number of meals, daily	76.5	79.4	97.1	84.3
Physical enrichment	100	94.1	94.1	96.1
Positive reinforcement training	94.1	82.4	79.4	85.3
Rearing history	100	85.3	76.5	87.3
Room cleaning frequency	88.2	88.2	91.2	89.2
Sedations, lifetime	91.2	82.4	79.4	84.3
See humans	79.4	70.6	73.5	74.5
See other non-human primates	73.5	91.2	82.4	82.4
Sensory enrichment	85.3	73.5	85.3	81.4
Social density	82.4	88.2	76.5	82.4
Social enrichment	94.1	91.2	97.1	94.1
Staff training	97.1	70.6	88.2	85.3
Surgeries, lifetime	97.1	85.3	94.1	92.2
Temperature of room	85.3	94.1	97.1	92.2
Timing of meals, daily	73.5	73.5	91.2	79.4
Ventilation	94.1	94.1	94.1	94.1
Vertical space	85.3	85.3	79.4	83.3
Vet med procedures, lifetime	82.4	76.5	79.4	79.4
Visual barrier, between caging	82.4	91.2	97.1	90.2
Visual barrier, within caging	82.4	88.2	82.4	84.3
Weaning age	85.3	76.5	76.5	79.4

The top animal-based indicators predominately focused on behaviours and health and appearance measures, whereas, for the environment-based indicators, the focus was on enrichment, environment, and management practice measures (Table 4).

**Table 4:** Top ten animal- and top ten environment-based indicators by composite percentage agreement score after Round Two.

Indicator type	Indicator description	Valid (%)	Reliable (%)	Feasible (%)	Composite score (%)
Animal-based	Self-harm behaviours	100	87.2	94.9	94.0
	Blood in waste	94.9	89.7	82.1	88.9
	Dyspnoea	94.9	82.1	89.7	88.9
	Stereotypical behaviours	82.1	87.2	97.4	88.9
	Huddled posture	89.7	76.9	92.3	86.3
	Appetite	92.3	79.5	82.1	84.6
	Injuries, NHP-induced	92.3	79.5	82.1	84.6
	Body weight	79.5	79.5	94.9	84.6
	Discharge	87.1	82.1	82.1	83.8
	Fear of NHPs	92.3	69.2^^^	89.7	83.7
Environment-based	Humane euthanasia program	100	94.2	100	98.1
	Health monitoring program	100	94.1	100	98.0
	Food enrichment	97.1	94.1	97.1	96.1
	Physical enrichment	100	94.1	94.1	96.1
	Social enrichment	94.1	91.2	97.1	94.1
	Ventilation	94.1	94.1	94.1	94.1
	Behavioural management program	100	88.2	94.1	94.1
	Hear other NHPs	88.2	91.2	97.1	92.2
	Cage furniture	97.1	91.2	88.2	92.2
	Temperature of room	85.3	94.1	97.1	92.2

^^^Indicates approaching agreement at a level of 65–69.99% agreement.

#### Ranking of welfare measures between rounds

For the top indicators in Round Two (Table 4), the inter-rater agreement (i.e. consensus) concerning the ranking of the top 20 indicators (10 animal- and 10 environment-based) selected from Round One, was good (W = 0.703 (*P* < 0.001)); however, there was some movement of items within Round Two (Table 5). Based on composite expert scores (n = 39) in Round Two, only five of the 10 animal-based indicators (50%) and nine of the 10 environment-based indicators (90%) from Round One were still considered valid, reliable, and feasible (Table 5) in Round Two. The remaining animal-based indicators were rated as less reliable (anxiety, body condition score, affiliative behaviours), less reliable or feasible (species-typical behaviour at abnormal levels), and less valid, reliable, or feasible (activity level), and so did not appear in the Round Two top indicators. For the remaining environment-based indicators, only qualifications/training of staff was not rated as valid, reliable, and feasible; four additional indicators (complexity of the cage/enclosure, daily observation by animal caregivers, cage/enclosure dimension, positive reinforcement training program) were considered as valid, reliable, and feasible, but dropped out of the top 10 highest ranked environment-based indicators based on composite scores (Table 5). Agreement about the ranking order of those indicators that were found in both Rounds One and Two improved between rounds.

**Table 5:** Expert ranking of welfare measures.

Indicator type	Indicator	Round One (n = 111)		Round Two, final (n = 39)		
		Group rank	Respondent agreement (%)	Group rank	Respondent agreement (%)	Composite score (%)
Animal-based	Self-harm behaviours*	1	60.2	1	94.9	94.0
	*Species-typical behaviour at abnormal levels*^*#*^	*7*	*19.5*	*2*	*69.2*	*63.2*
	Appetite*	4	36.3	4	64.1	84.6
	*Anxiety behaviours^*	*3*	*41.6*	*4*	*64.1*	*80.3*
	*Body condition score^*	*9*	*15.9*	*4*	*64.1*	*77.8*
	*Affiliative behaviours^*	*6*	*26.6*	*6.5*	*61.5*	*73.5*
	*Activity level*^*##*^	*8*	*17.7*	*6.5*	*61.5*	*56.4*
	Stereotypical behaviours*	2	46.0	8	59.0	88.9
	Injuries, NHP-induced*	5	31.9	9	53.8	84.6
	Body weight*	9	15.9	10	38.5	84.6
Environment-based	Social enrichment*	1	54.0	1	94.9	94.9
	Complexity of the cage/enclosure*	*10*	*19.5*	*2*	*66.7*	*80.3*
	Behavioural management program*	2	42.5	3	64.1	93.2
	Daily observation by animal caregivers*	*3*	*39.8*	*4*	*61.5*	*88.9*
	Cage/enclosure dimension*	*9*	*22.1*	*5*	*59.0*	*84.6*
	Positive reinforcement training program*	*4*	*25.7*	*6*	*56.4*	*86.3*
	Health monitoring program*	6	24.8	7	48.7	97.4
	Food enrichment*	4	25.7	8.5	23.1	96.6
	*Qualifications/ training of staff^*	*6*	*24.8*	*8.5*	*23.1*	*70.1*
	Physical enrichment*	6	24.8	10	17.9	96.6

Italics = indicators eliminated from experts’ top 10 between rounds one and two.

*Valid, reliable, and feasible.

^Less reliable.

^#^Less reliable or feasible.

^##^Less valid, reliable, or feasible.

#### Welfare measures by indictor type

Indicator type influenced response selection (Table 6); specifically, environment-based indicators were selected more across rounds One and Two than animal-based indicators. A binomial test indicated that the proportion of animal-based indicators of 0.47 was lower than the expected 0.51, *P* < 0.001 (1-sided)*.* Back-transformed means in this model again confirm that the responses between rounds remained stable. Additionally, respondents found indicators to be valid more than they did feasible or reliable.

**Table 6:** Selection of animal- and environment-based indicators across rounds One and Two: GLMM 2 [round, indicator type, response type (fixed effects); respondent (random effect)].

	Mean	Back-transformed mean	SEM (avg)	SEM (lower)	SEM (upper)
**Round**					
1	0.8540	0.7014	0.01933	0.01916	0.01951
2	0.9421	0.7195			
**Indicator type**					
Animal-based	0.5249	0.6283	0.01935	0.01845	0.02026
Environment-based	1.2712	0.7809			
**Response type**					
Valid	1.0623	0.7431	0.02367	0.02307	0.02448
Reliable	0.7727	0.6841			
Feasible	0.8590	0.7025			

## Discussion

The aim of this study was to identify and determine the relative value of different potential measures of laboratory-housed macaque welfare through expert consultation about the validity, reliability, and feasibility of the measures. The overall level of consensus reached by the experts as to 115 measures that should be used to assess macaque welfare based on their validity, reliability, and feasibility was 67.5%. This was just below the pre-determined level of 70% agreement necessary for consensus as applied in other welfare studies^21,54,55^. Attempting to reach ≥ 70% consensus on all 115 indicators over three factors was always going to be a challenge and is more complex than other studies in other contexts^21,55^. As such, the consensus of 67.5% was deemed sufficient for this study as important insight was gained in breaking down the indicators into categories^55^. For almost half of the indicators (n = 57), consensus was approached (65–69.99%) or there was mixed agreement/dissensus (see Supplementary Table S4 online); this is likely due to a combination of factors including the specific indicator, the supplied on-site assessment scenario within the survey instrument, and differences in the demographics of the experts. A third round was not pursued as consensus (67.5%) was only just short of the predetermined level of 70% for the 115 indicators. The diminished rate of return for the second round (n = 72) was more than twice what was expected, suggesting that an additional round would result in too few respondents for any relevant analysis. Nonetheless, there were enough respondents in Round Two (n = 39) to reach reliable consensus^56^. This is further supported by the relatively high group stability observed between rounds, serving as a secondary criterion for termination of the iterative process^48,57^. The responses of the experts were generally consistent, both as individual (i.e. within an expert) and as a group (i.e. between experts), leading to high between-round stability. This could be either due to the group feedback provided from Round One inducing little change in their responses in Round Two, i.e. they remained firm in their original Round One choices despite the feedback, or that respondents ignored the feedback from Round One, which would also lead to round stability.

The group agreed that environment-based measures of welfare are better suited for on-site assessment than animal-based ones. Although animal-based measures were considered as valid, experts did not consider as many of them to be either as reliable or feasible to measure (see Supplementary Table S4 online), echoing the difficulties found in practically using them in welfare assessment protocols^58^. The European Food Safety Authority’s Panel on Animal Health and Welfare^17^ recommends assessing validity (i.e. whether the indictor measures and reflects a welfare outcome) of animal-based welfare indicators via study-based validation, which has not been completed for most in use for macaques as evidenced by the dearth of literature on the topic, or by expert opinion, as done in this study. The experts reaching consensus concerning the validity, reliability and/or feasibility of the 115 indicators presented (animal- and environment-based) in this study implies that these indices can now be used as a form of benchmark. Other indices that are used currently for welfare assessment but have yet to be validated or novel indices that have not been used can be compared against the indices identified in this study, for example some of the animal-based items listed on the NC3Rs website on macaques^59^.

Observable behaviour, an animal-based indicator, is most typically used to assess macaque welfare^60^, as well as the welfare of other laboratory-housed animals^61^, because of its ease in collection (i.e. feasibility). Furthermore, the expression of abnormal behaviour, which includes stereotypical/abnormal repetitive and self-harm behaviours, among others, is thought to reflect poor welfare as it is either pathological or associated with environmental coping^36,62^ and so is often used as a proxy for welfare^61,63,64^. However, many types of observable behaviour are yet to be validated as a means of assessing welfare and are only now being empirically explored to define their role in macaque welfare assessment (e.g. hair-loss as a biomarker for stress^65^).

The results of this study serve to narrow the field of indices requiring validation, lend some credence to those currently used to measure welfare within the laboratory (e.g. abnormal behaviour), and highlight indices that are not considered effective for welfare assessment. For example, telomere length was specifically rejected as experts agreed that it is not valid, reliable, or feasible to measure within a half-day site visit. Further, this Delphi study can be viewed as a starting point for eventual scientific assessment of macaque welfare, as has been done in similar studies with other captive species, like commercial finishing pigs^49,66^ and laboratory mice^21,22,67^.

In addition to confirming potential indicators, experts were asked to place a relative value on them. Experts were asked to rank the top ten most important animal and environmental indicators for welfare assessment without guidance (i.e. based on validity, reliability, or feasibility). Across rounds, experts agreed that self-harm behaviours and provision of social enrichment are the most important indicators for assessing macaque welfare. These are in-line with the focus of research publications specific to laboratory-housed macaques, including on how to minimize or treat self-harm behaviours^35,36,68–74^, and the importance of social housing^12,75,76^, and associated techniques^77,78^ and so emphasising the utility of these findings. Agreement of the ranking of each item improved between rounds; however, this could be attributed to a smaller sample in Round Two or to the composition of the panel. Heterogeneity of a group is thought to lead to better results within a group decision-making process^47^; however, nearly half of those completing both rounds were employed in behavioural management or animal welfare positions. It is likely that those who opted to participate in each survey round not only have a vested interest in the finished product in their occupation (i.e. a list of macaque welfare indicators), but also share similar selection criteria for indicator ratings. The composite score percentage agreement of the items identified as the top welfare measures (Table 5) indicates dissensus as to the order of their importance. For example, activity level, included in the ranking of welfare measures from Round One, was rejected in Round Two as not valid, reliable, or feasible. Body weight, an indicator deemed valid, reliable, and feasible, is ranked 10th most important as an animal-indicator, but there is disagreement as to where it should rank as only 38.5% of experts agree to its positioning. Other items were less reliable or both less reliable and less feasible, suggesting that validity was the primary consideration in the ranking of items. The top welfare indicators by composite percentage agreement score (Table 4) indicate that reliability is a concern for experts more so than feasibility and validity (i.e. percentage agreement scores are lower for reliability) with both indicator types; this may be related to the subjective judgements involved with observer ratings while conducting assessments.

While observer ratings have been widely used for many types of research and can be practical to implement (e.g. welfare monitoring in zoos^79^; QBA of sheep^80^), they can be influenced by knowledge and experience^61^ and subject to expectation bias, in which an opinion is shaped by non-task-related information especially confirmatory information^81^. For example, if a caretaker is asked to report the occurrence of abnormal behaviours in an individual, they might spend more time observing that animal than in their normal routine, looking for any occurrence; a newly trained caretaker might report more types and higher occurrences of such behaviours than a seasoned individual because of uncertainty in what they are observing. This bias, along with fear of anthropomorphism and the reliance of interpretation on an animal’s experience^82^, may be why there is hesitancy to implement and draw conclusions from observer ratings in some circumstances, such as on-site welfare assessment. However, observer ratings are unavoidable if relevant welfare indicators, particularly behavioural ones, are to be included in a comprehensive assessment tool. To be useful in an on-site assessment, ratings must be valid, reliable, and feasible. Reliability, the extent to which a measurement is repeatable and consistent (reproducible), hinges upon operationally defining measurement techniques, and adequately defining what it is that is being measured, both of which can impact inter-observer and test–retest reliability^83^. For example, detailed scoring systems with multiple classes can pose reliability issues as there are more opportunities for disagreement in scoring; collapsing classes where possible could alleviate reliability issues, but risks elimination of data that might be helpful in discriminating between levels of welfare^84^. Nevertheless, scoring systems, like those used to measure alopecia^85,86^ and body condition^87,88^ in macaques, can be successfully implemented as along as inter- and intra-observer reliability are regularly assessed. Indicator usefulness will be determined by whether people can use it to assess welfare, despite difficulties; hence the importance for empirical-based evaluations that explore and define the potential limitations of each for on-site assessment.

There was little difference in the number of parameters offered for rating between the two indicator types, yet experts selected more than three times the number of environment-based input measures (72%) as valid, reliable, and feasible for on-site welfare assessments compared to the animal-based output measures (22%). There may be several reasons for this based on the characteristics of each indicator type. Although environmental input parameters have the potential for low validity since they are indirect measures of welfare and can be experienced differently by the individual, they are typically easier to measure (i.e. more feasible) and can be more reliably measured between raters^43^. For example, measuring temperature of a room is simple enough—it requires little time to measure, is low cost because of no associated training or extra equipment, and can be measured repeatedly across raters and visits. In contrast, even though outcome or performance measures assessed directly from the animal, like behavioural or health measures, are likely to reflect the actual welfare state of the individual^17^, they are often time-consuming to assess, pose reliability problems, and can be impractical if difficult to measure, especially when trained personnel are required to gather data (e.g. veterinary personnel to sample blood). If, for instance, an assessor was interested in macaque hair loss, they would have to either score all or a sample of the population of the animals or rely on in-house records, if they exist. Next, they would need to address temporal considerations (e.g. when did the hair loss occur?) and factors associated with data collection (e.g. are personnel adequately trained? have behavioural and/or veterinary courses of action been pursued for causality and treatment?). Finally, they would need to contextualize the welfare indicator (e.g. is the hair loss associated with a research study that typically results in hair loss or is it due to over-grooming in a social pair?). Identifying welfare indicators is the first step in providing scientific-based guidance for managing perceived welfare issues; clearly, validation to simplify some of this process, especially for animal-based indicators, is needed.

The ability of the environment-based measures to be implemented quickly to a large population of animals (i.e. large colony) is of particular importance for laboratory animals such as macaques. Unlike other captive environments like zoos and sanctuaries, laboratories sometimes house more primates, and individuals can be found in a range of housing types, such as outdoor corrals, indoor-outdoor runs, or indoor caging; assessing these populations in a day or less poses challenges similar to farm assessments, like implementation of animal-based indicators. Although, a population size was provided in the scenario for the survey, optimal sampling sizes and observation periods for each indicator were not, as they have yet to be established. Establishing these via a Delphi process, as Leach and colleagues^21^ did in their study identifying assessment measures of welfare for laboratory mice, could drastically alter respondent answers. If respondents could indicate validity, reliability, and feasibility within the context of multiple sampling scenarios, this might be more informative than the approach taken in this study and might reveal the scenarios in which animal-based indicators are preferred.

To effectively evaluate the present welfare status of an animal and measure improvement of that state over time based on any management interventions, it is important that all components of welfare be measured and in a meaningful way. This study provides an empirical basis upon which to start the validation of indicators that can be integrated into assessment tools developed for macaques and emphasizes the need to include both environment- and animal-based indicators in any such tools for accurate welfare monitoring. This study provides guidance on the next steps for developing a tool to help ensure good welfare, rather than just meeting minimum standards of care. Expert respondents have provided a list of animal- and environment-based items considered valid, reliable, and feasible for on-site assessment, most of which need to undergo empirical assessment in a variety of captive environments (e.g. laboratories, zoos, sanctuaries). These indicators may be helpful to zoos, for example, as they could be integrated into existing tools for assessing smaller populations of macaques (e.g. Detroit Zoological Society Individual Animal/Environment Welfare Assessment^89^). Application of the Delphi consultation process with zoo employees and stakeholders in other captive environments could be beneficial so that cross-environment indicators can be identified and validated; this is of particular importance as more laboratory NHPs are retired and move to different surroundings. Once validation is undertaken, development of a comprehensive welfare assessment tool, one that includes negative and positive measures of welfare, can be explored.

## Methods

**Figure 1:**
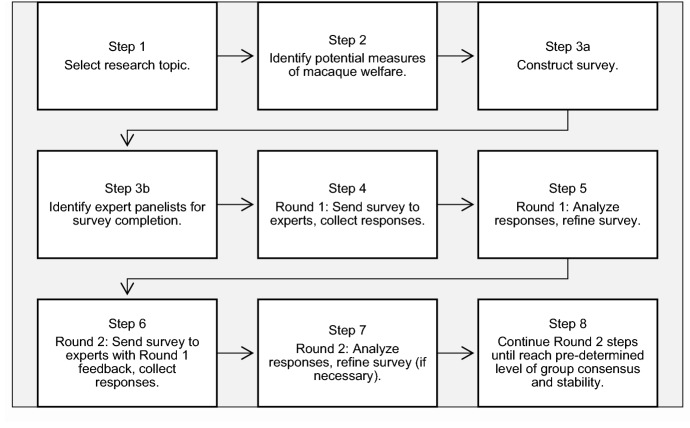
Steps in a modified Delphi process.

The modified Delphi consultation process was completed using steps illustrated in Fig. 1.

### Ethical consideration

Data collection procedures were approved by the Human Ethics Research Committee, University of Edinburgh (approval #HERC_157_17). Due to the iterative nature of the Delphi consultation process (i.e. the need to tie responses to users to provide individualized feedback), quasi-anonymity was maintained—responses remained unknown to other participants but were known to the researchers. However, to maximize anonymity, response data were coded by username after receipt so that individuals’ responses could not be readily linked and identifying information and data results were kept separate always. All data were handled and stored in compliance with the UK Data Protection Act 1998.

### Identification of initial list of indicators

A list of 115 potential measures of laboratory-housed macaque welfare was generated using multiple literature searches on Web of Science between January 1965 and August 2017; English language results were used to search abstract content and titles. A total of 709 unique results were yielded from the following keywords and phrases: health, macaque(s), primate(s), macaca, welfare, well-being, P(sychological)W(ell)B(eing), alopecia, quality of life; ape(s), orangutan(s), and chimp(anzee)(s) were excluded. Potential welfare indicators were selected if an item was related to the welfare, quality-of-life, or well-being of macaques. Items related to environmental enrichment, housing, and health and management practices were categorized as environment-based (input) measures; those related to the animals’ appearance and physical health and the behavioural and physiological response to the environment were categorized as animal-based (output) measures (see Fig. 1, Steps 1–2).

The initial list of 115 potential indicators comprised 61 environment-based and 54 animal-based items (Tables 7 and 8).

**Table 7:** Initial list of potential environment-based indicators.

Enrichment	Environment	Health and management practices
Access to exercise/play area	Auditory access to neighbouring conspecifics	Behavioural management program
Browse provision	Cage/enclosure dimension	Blunting of canine teeth
Frequency of exposure to novel items, intentional (e.g. toys)	Cage/enclosure furniture (e.g. swings, ladders, perching)	Daily observation by animal caregivers
Frequency of exposure to novel items, unintentional (e.g. new uniform)	Clear visual access to approaching humans	Disease surveillance & diagnosis
Multiple manipulanda in/on caging/enclosure	Clear visual access to neighbouring conspecifics	Frequency of handling by humans: chair restraint
Positive reinforcement training (PRT) program	Complexity of the cage/enclosure	Frequency of handling by humans: hand-catching
Provision of cognitive/occupational enrichment (e.g. computerized tasks, exercise opportunities)	Escape-proof enclosures (e.g. self-closing doors)	Health monitoring program
Provision of destructible enrichment (e.g. cardboard, paper, wood)	Exterior windows to hallways or outdoors	Humane euthanasia procedure
Provision of food enrichment	Flooring type	Inoculation history per lifetime
Provision of materials for thermoregulation	Frequency of enclosure/room cleaning procedures	Meals per day, number
Provision of natural materials in housing	Humidity, room	Meals per day, timing
Provision of physical enrichment	Increased field of view (e.g. provision of cage extension/porch, mirror)	Number of moves within/between caging/housing areas per lifetime
Provision of sensory (visual, auditory, tactile, gustatory, olfactory) enrichment	Intensity of lighting	Number of sedations/anesthetizations per lifetime
Provision of social enrichment	Light source (fluorescent, natural)	Number of surgical procedures (major, minor) per lifetime
Substrate type	Noise levels	Number of times participated in an experiment per lifetime
Variety of enrichment food types	Position of the caging in the room	Number of veterinary procedures per lifetime
	Presence of vibration	Qualifications/training of staff
	Social density	Quality of life assessments
	Social stability	Rearing history
	Spatial density	Weaning age
	Temperature, room	
	Ventilation, room	
	Vertical space	
	Visual barrier(s), between caging	
	Visual barrier(s), within caging

**Table 8:** Initial list of potential animal-based indicators.

Appearance and health measures	Behaviour	Physiology and genetics
Alopecia score	Abuses/neglects infant	Acute phase proteins
Ambulation/gait	Activity level	Blood pressure
Appetite	Affiliative behaviour with conspecific(s)	Body temperature
Atrophy	Aggressive behaviour with conspecific(s)	Body weight
Blood in urine/stool	Anxiety behaviour	Cortisol concentration
Body condition score	Cagemate(s) behaviour towards individual	Genotype
Coat condition	Decreased maintenance behaviours	Heart rate
Coughing	Excessive fear of or withdrawal from conspecifics	Heterophil: lymphocyte ratio
Discharge, ocular/nasal	Facial expression, changes in	Lymphocyte activity
Dyspnoea (laboured breathing)	Huddled posture	Respiration rate
Fatigue/lethargy	Neophobia	Telomere length
Fertility/Ability to produce offspring for non-sterilized individual	Overgroom/hair pluck of cagemate(s)	
Growth/development rate	Piloerection	
Hydration status	Play	
Injuries, environmentally induced	Reaction to human approach: Aggressive	
Injuries, self- or cagemate-induced (e.g. bite wound)	Reaction to human approach: Fearful	
Morbidity rate	Self-harm behaviours	
Mortality rate	Species-typical behaviour at abnormal levels	
Number of diarrhoea diagnoses per lifetime	Stereotypical/abnormal repetitive behaviours	
Prolapse	Vocalizations	
Prostration		
Urination, excessive or lack of		
Water intake		

### Panel formation

The aim was to purposively sample approximately 400 qualified persons to meet the set response rate of 25% for Round One (n = 100), adequate for a Delphi survey^49^. The rate of attrition between Delphi rounds is reported at 30%^90^; this would leave 70 potential respondents for a second round, more than the 25–60 needed to reach reliable consensus^56^. A relatively poor response rate in a Delphi process is expected because of its iterative nature^46,91^.

Concurrent with survey construction, a research panel was formed. The panel was comprised of participants with expertise in one or more of the following fields as they pertain to captive *Macaca*: veterinary medicine, behavioural management/animal welfare, animal husbandry, facility management, and research. For inclusion, panellists had to be 18 years or older and have more than one year of experience working with or studying one or more macaque species. Purposive and snowball sampling resulted in a total of 477 panellists that were asked to participate. Prospective respondents were identified through authorship of the literature reviewed for potential indicators, the professional network of the researchers, and employment of a snowballing technique^92^ (Fig. 1, Step 3b).

### Data collection

#### Survey—Round One: piloting and finalization

The survey was created using the Bristol Online Survey (BOS) software (Jisc 2017), and consisted of multiple sections: project information and participant consent request; demographics questions to establish subject eligibility; the rating of macaque welfare indicators; and the selection of indicators viewed as the most important for macaque welfare assessment. The survey was reviewed in a two-part piloting phase by 12 persons that included both laypersons and non-macaque captive NHP experts. This pilot panel ensured face and content validity of indicators, the appropriateness of the questionnaire items in relation to the study aims, and that the survey was properly categorized, organized, functional, clear, for an approximate completion time of 25 min. Pilot test phase respondents did not serve as survey respondents; their feedback was incorporated in the version of the survey created for Round One distribution (see Supplementary Fig. S5 online for example of Round One survey).

#### Survey—Round One

Two versions of the Round One survey were created for randomized equal distribution between the potential respondents to minimize response order effects; the order of the environment-based and animal-based items were swapped; the surveys were otherwise identical (Fig. 1, Step 3a).

Initially, demographic questions were asked relating to *Macaca* experience, occupation, education, age, and country of residence. This was then followed by participants being asked to rate the 115 potential indicators provided as valid, reliable, and feasible (or not). They could also select “undecided” when considering each measure and add missing indicators (if desired). These questions were asked within the context of the following half-day welfare assessment scenario:

‘Assume that you are participating in a welfare audit in an institution housing approximately 500 macaques. Individuals are housed indoors in 25 animal rooms which each hold 5 racks; each rack holds 4 cages and each cage houses 1 monkey. Monkeys are either singly housed with access to one cage or are socially housed in pairs or groups with access to multiple adjacent cages (1 per animal) within a single rack; some individuals are participating in active research studies’.

The participants were then asked to choose a total of ten animal and ten environmental indicators they thought most important for assessing macaque welfare from the provided list of 115 items; they were not given guidance in how to select these (e.g. the most valid or the most feasible). Definitions were provided for these terms: welfare, indicator, valid, reliable, and feasible.

One-hundred fourteen respondents from eight countries (Canada, England, France, Germany, Netherlands, South Africa, Taiwan, USA) completed the survey (24% response rate) between the allotted period, 17 January to 7 February 2018. Three responses were discarded as two respondents did not meet inclusion criteria and one withdrew (Fig. 1, Step 4). Responses were analysed to compile response feedback and the survey was refined for Round Two (Fig. 1, Step 5).

#### Survey—Round Two

For the second round of the consultation process, an electronic survey was created using Microsoft Excel (2016) and distributed electronically to the 111 qualified participants who completed the first-round survey. Each participant received a personalized survey (see Supplementary Fig. S6 online for example of Round Two survey) based on the results of Round One that included their responses to the questions posed, the combined responses of the group, presented as respondent percentage agreement (i.e. controlled feedback, Fig. 1, Step 6), and the ten measures most selected by respondents from both the animal- and environment-based indicators in the form of group agreement (%) and each indicator’s rank position. Participants were initially given the opportunity to alter their choices (or not) relating to the 115 potential welfare indicators from Round One, in terms of their validity, reliability, and feasibility in the context of the same hypothetical scenario (described in Round One), and to re-rank the top ten animal- and top ten environment-based indicators if they disagreed with the presented order from Round One.

A total of 39 surveys were returned (35% response rate) in the provided response time, 18 February to 11 March 2018. Participants were from Canada, France, South Africa, and the United States. Responses were analysed to determine whether the group had reached consensus and response stability on the presented indicators; this informed whether a third round was necessary (Fig. 1, Steps 7–8).

### Statistical analyses

Statistical analyses were generated by SPSS (IBM SPSS Statistics, version 22.0 2013; IBM Corp, Armonk, NY, USA) and GenStat (GenStat for Windows, 19th edition 2017; VSN Intl, Hemel Hempstead, UK) statistical packages, and Excel 2016 for graphical output. Non-parametric statistical procedures were used due to the relatively small sample size and ordinal data, with a set significance value of *P* < 0.05. Percentage agreements were calculated to supplement each statistical test. The mean of the validity, reliability, and feasibility percentage agreement scores was calculated for each indicator to provide a composite respondent agreement score.

The indicator scoring scales consisted of categorical, ordinal data. Scores were dichotomized into agree (valid/reliable/feasible) and disagree (not valid/reliable/feasible and undecided) for analysis. Ranked ordinal data were not dichotomized.

For binary scores, multiple generalized linear mixed models (GLMMs) were used to assess the differently distributed responses sampled by group (i.e. the same respondents over two rounds) and to account for both random and fixed effects. Multiple GLMM regressions with a binomial distribution were run (see Supplementary Fig. S7 online); all included unique respondent number (UserID) as a random effect since the data were paired between rounds. Round was included as a fixed effect in each model, as were other variables (e.g. indicator, indicator type, response type, UserID) dependent on the question of interest.

Krippendorff’s alpha coefficient (α) test^93^ was employed to test group stability of respondents. For interpretation, a value of 0 indicates perfect disagreement whereas 1 indicates perfect agreement; a value of 0.667 or more permits (tentative) conclusions to be made^94^.

Agreement between raters on the ranking of the top ten animal- and environment-based indicators was assessed using Kendall’s coefficient of concordance (W); a value of 0 indicates no agreement, less than or equal to 0.30 weak agreement, 0.31–0.50 moderate, 0.51–0.70 good, 0.71–0.99 strong, and 1 perfect agreement^95^.

## Supplementary information

Supplementary Information

## Data Availability

The datasets generated during and/or analysed during the current study are not publicly available due to compliance with General Data Protection Regulation (EU) 2016/679 (GDPR) but are available from the corresponding author on reasonable request.
