# Supplementary material for: The identification of effective welfare indicators for laboratory-housed macaques using a Delphi consultation process
Source: Sci Rep. 2020 Nov 23;10:20402. doi: 10.1038/s41598-020-77437-9 (PMC7683591; doi:10.1038/s41598-020-77437-9)
Supplement: Supplementary file 1 — Supplementary Information [file 41598_2020_77437_MOESM1_ESM.docx]

**The Identification of Effective Welfare Indicators for Laboratory-Housed Macaques using a Delphi Consultation** **Process**

Melissa A. Truelove^1,2^, Jessica E. Martin^2^, Fritha M. Langford^2,3^, and Matthew C. Leach^4^

^1^Yerkes National Primate Research Center, Emory University, Atlanta, Georgia, USA

^2^ Royal (Dick) School of Veterinary Studies and the Roslin Institute, University of Edinburgh, Edinburgh, UK

^3^Animal and Veterinary Sciences, SRUC, West Mains Road, Edinburgh EH9 3JG, UK

^4^School of Natural and Environmental Sciences, Newcastle University, Newcastle-Upon-Tyne, UK

**Supplementary Information**

Respondent demographics, example surveys for rounds one and two, xxx are shown below.

Tables S1-S3 Respondent demographics.

**Supplementary Table S1.** Participant age, location, and education.

| Age | N | Respondents (%) |
| --- | --- | --- |
| 18-24 | 0 | 0 |
| 25-34 | 5 | 12.8 |
| 35-44 | 19 | 48.7 |
| 45-54 | 8 | 20.5 |
| 55-64 | 6 | 15.4 |
| 65 and over | 1 | 2.6 |
| Region |  |  |
| Africa | 1 | 2.6 |
| Asia | 0 | 0 |
| Australia | n/a | n/a |
| Europe | 3 | 7.7 |
| North America | 35 | 89.7 |
| South America | n/a | n/a |
| Education |  |  |
| High school degree or less | 0 | 0 |
| Some college or Associate's degree | 4 | 10.3 |
| Bachelor’s degree | 6 | 15.4 |
| Master’s degree | 10 | 25.6 |
| Professional degree | 4 | 10.3 |
| Doctorate | 15 | 38.5 |
| Coursework in animal welfare |  |  |
| Yes | 24 | 61.5 |
| No | 15 | 38.5 |

**Supplementary Table S2.** Occupation and employment type.

| Current occupation | n | Respondents (%) |
| --- | --- | --- |
| Animal caregiver | 0 | 0 |
| Animal caregiver supervisor or manager | 1 | 2.6 |
| Behavioural management or animal welfare technician | 5 | 12.8 |
| Behavioural management or animal welfare supervisor or manager | 6 | 15.4 |
| Behavioural management or animal welfare scientist | 6 | 15.4 |
| Colony management | 0 | 0 |
| Researcher or research staff | 12 | 30.8 |
| Veterinarian or veterinary technician | 7 | 18 |
| Other | 2 | 5.1 |
|  |  |  |
| Current occupation, tenure |  |  |
| Less than 1 year | 2 | 5.1 |
| 1-5 years | 5 | 12.8 |
| 6-10 years | 8 | 20.5 |
| Over 10 years | 24 | 61.5 |
|  |  |  |
| Primary employer type |  |  |
| Academic | 9 | 23.1 |
| Consultancy | 2 | 5.1 |
| Contract research organization | 2 | 5.1 |
| Pharmaceutical | 0 | 0 |
| Primate breeding facility | 2 | 5.1 |
| Primate research centre | 16 | 41 |
| Regulatory | 0 | 0 |
| Sanctuary | 1 | 2.6 |
| Zoological park | 1 | 2.6 |
| Other | 6 | 15.4 |

**Supplementary Table S3.** Experience with macaques.

| Location of experience | n | Respondents (%) |
| --- | --- | --- |
| Captive | 34 | 87.2 |
| Wild | 0 | 0 |
| Captive and wild | 5 | 12.8 |
|  |  |  |
| Years of experience |  |  |
| 1 to 5 years | 4 | 10.3 |
| 5 to 10 years | 6 | 15.4 |
| 10 years or less | 10 | 25.6 |
| Over 10 years | 29 | 74.4 |
|  |  |  |
| Species |  |  |
| Cynomolgus (*M. fascicularis*) | 31 | 79.5 |
| Rhesus (*M. mulatta*) | 39 | 100 |
| Pigtail (*M. nemestrina*) | 20 | 51.3 |
| Bonnet (*M. radiata*) | 4 | 10.3 |
| Japanese (*M. fuscata*) | 5 | 12.8 |
| Stump-tailed (*M. arctoides*) | 5 | 12.8 |
| Other | 4 | 10.3 |

**Supplementary Table S4.** Respondent percentage agreement of the 115 welfare indicators by response type.

| Indicator Type | Indicator | Indicator Description | Valid (%) | Reliable (%) | Feasible (%) | Composite Score (%) |
| --- | --- | --- | --- | --- | --- | --- |
| Animal-based | 1 | Alopecia | 43.6 | 51.3 | 92.3 | 62.4 |
|  | 2 | Ambulation/gait | 89.7 | 69.2^^^ | 84.6 | 81.2 |
|  | 3 | Appetite | 92.3 | 79.5 | 82.1 | 84.6 |
|  | 4 | Atrophy | 74.4 | 69.2^^^ | 61.5 | 68.4 |
|  | 5 | Blood in waste | 94.9 | 89.7 | 82.1 | 88.9 |
|  | 6 | Body condition score | 76.9 | 69.2^^^ | 87.2 | 77.8 |
|  | 7 | Coat condition | 82.1 | 35.9 | 89.7 | 69.2 |
|  | 8 | Coughing | 74.4 | 61.5 | 64.1 | 66.7 |
|  | 9 | Discharge | 87.1 | 82.1 | 82.1 | 83.8 |
|  | 10 | Dyspnoea | 94.9 | 82.1 | 89.7 | 88.9 |
|  | 11 | Fatigue | 92.3 | 48.7 | 64.1 | 68.4 |
|  | 12 | Fertility | 28.2^*^ | 35.9 | 30.8 | 31.6 |
|  | 13 | Growth rate | 71.8 | 64.1 | 66.7^^^ | 67.5 |
|  | 14 | Hydration | 92.3 | 71.8 | 59.0 | 74.4 |
|  | 15 | Injuries, environmental | 84.6 | 71.8 | 82.1 | 79.5 |
|  | 16 | Injuries, NHP | 92.3 | 79.5 | 82.1 | 84.6 |
|  | 17 | Morbidity | 84.6 | 69.3^^^ | 71.8 | 75.2 |
|  | 18 | Mortality | 79.5 | 82.1 | 79.5 | 80.3 |
|  | 19 | Diarrhoea diagnoses | 69.2^^^ | 71.8 | 82.1 | 74.4 |
|  | 20 | Prolapse | 71.8 | 71.8 | 76.9 | 73.5 |
|  | 21 | Prostration | 59.0 | 59.0 | 71.8 | 63.2 |
|  | 22 | Urination | 69.2^^^ | 56.4 | 35.9 | 53.8 |
|  | 23 | Water intake | 66.6^^^ | 56.4 | 33.3 | 52.1 |
|  | 24 | Abuses infant | 51.3 | 48.7 | 64.1 | 54.7 |
|  | 25 | Activity | 69.2^^^ | 46.2 | 53.8 | 56.4 |
|  | 26 | Affiliation | 92.3 | 56.4 | 71.8 | 73.5 |
|  | 27 | Aggression | 87.2 | 61.5 | 71.8 | 73.5 |
|  | 28 | Anxiety | 87.2 | 66.6^^^ | 87.2 | 80.3 |
|  | 29 | Cagemate behaviour | 69.2^^^ | 43.6 | 61.5 | 58.1 |
|  | 30 | Self-maintenance behaviours | 84.6 | 51.3 | 51.3 | 62.4 |
|  | 31 | Fear of NHPs | 92.3 | 69.2^^^ | 89.7 | 83.8 |
|  | 32 | Facial expressions | 41.0 | 23.2^*^ | 35.9 | 33.3 |
|  | 33 | Huddled posture | 89.7 | 76.9 | 92.3 | 86.3 |
|  | 34 | Neophobia | 35.9 | 33.3 | 69.2^^^ | 46.2 |
|  | 35 | Overgroom | 43.6 | 51.3 | 69.2^^^ | 54.7 |
|  | 36 | Piloerection | 28.2^*^ | 35.9 | 61.5 | 41.9 |
| Animal-based | 37 | Play | 76.9 | 56.4 | 64.1 | 65.8 |
|  | 38 | Aggression to human | 43.6 | 46.3 | 82.1 | 57.3 |
|  | 39 | Fear of human | 66.6^^^ | 51.3 | 82.1 | 66.7 |
|  | 40 | Self-harm behaviours | 100.0 | 87.2 | 94.9 | 94.0 |
|  | 41 | Abnormal level species-typical behaviours | 79.5 | 53.8 | 56.4 | 63.2 |
|  | 42 | Stereotypical behaviours | 82.1 | 87.2 | 97.4 | 88.9 |
|  | 43 | Vocalizations | 30.8 | 20.5^*^ | 51.3 | 34.2 |
|  | 44 | Acute phase proteins^**^ | 15.4^*^ | 20.5^*^ | 28.2^*^ | 21.4 |
|  | 45 | Blood pressure | 66.6^^^ | 59.0 | 23.1^*^ | 49.6 |
|  | 46 | Body temperature | 69.2^^^ | 69.2^^^ | 43.6 | 60.7 |
|  | 47 | Body weight | 79.5 | 79.5 | 94.9 | 84.6 |
|  | 48 | Cortisol concentration | 61.5 | 59.0 | 38.5 | 53.0 |
|  | 49 | Genotype | 18.0^*^ | 38.5 | 20.5^*^ | 25.6 |
|  | 50 | Heart rate | 64.1 | 56.4 | 33.3 | 51.3 |
|  | 51 | H/L ratio | 30.8 | 28.2^*^ | 28.2^*^ | 29.1 |
|  | 52 | Lymphocyte activity | 33.3 | 25.6^*^ | 28.2^*^ | 29.1 |
|  | 53 | Respiration rate | 64.1 | 61.5 | 48.7 | 58.1 |
|  | 54 | Telomere length^**^ | 15.4^*^ | 20.5^*^ | 12.8^*^ | 16.2 |
| Environment-based | 55 | Access to exercise/play space | 79.4 | 76.5 | 64.7 | 73.5 |
|  | 56 | Browse provision | 88.2 | 76.5 | 91.2 | 85.3 |
|  | 57 | Novelty exposure, intentional | 85.3 | 82.4 | 97.1 | 88.3 |
|  | 58 | Novelty exposure, unintentional | 23.4^*^ | 23.5^*^ | 55.9 | 34.3 |
|  | 59 | Manipulanda | 88.2 | 79.4 | 94.1 | 87.2 |
|  | 60 | Positive reinforcement training | 94.1 | 82.4 | 79.4 | 85.3 |
|  | 61 | Cognitive enrichment | 94.1 | 79.4 | 61.8 | 78.4 |
|  | 62 | Destructible enrichment | 94.1 | 82.4 | 88.2 | 88.2 |
|  | 63 | Food enrichment | 97.1 | 94.1 | 97.1 | 96.1 |
|  | 64 | Thermoregulation materials | 58.8 | 47.1 | 55.9 | 53.9 |
|  | 65 | Natural materials | 64.7 | 76.5 | 67.6^^^ | 69.6 |
|  | 66 | Physical enrichment | 100.0 | 94.1 | 94.1 | 96.1 |
|  | 67 | Sensory enrichment | 85.3 | 73.5 | 85.3 | 81.4 |
|  | 68 | Social enrichment | 94.1 | 91.2 | 97.1 | 94.1 |
|  | 69 | Substrate type | 58.8 | 64.7 | 52.9 | 58.8 |
|  | 70 | Food variety | 88.2 | 82.4 | 97.1 | 89.2 |
| Environment-based | 71 | Hear other NHPs | 88.2 | 91.2 | 97.1 | 92.2 |
|  | 72 | Cage dimension | 79.4 | 85.3 | 88.2 | 84.3 |
|  | 73 | Cage furniture | 97.1 | 91.2 | 88.2 | 92.2 |
|  | 74 | See humans | 79.4 | 70.6 | 73.5 | 74.5 |
|  | 75 | See other NHPs | 73.5 | 91.2 | 82.4 | 82.4 |
|  | 76 | Cage complexity | 88.2 | 79.4 | 73.5 | 80.4 |
|  | 77 | Escape-proof enclosures | 64.7 | 67.6^^^ | 76.5 | 70.6 |
|  | 78 | Window access | 61.8 | 70.6 | 67.6^^^ | 66.2 |
|  | 79 | Flooring type | 47.1 | 67.6^^^ | 67.6^^^ | 47.1 |
|  | 80 | Room cleaning frequency | 88.2 | 88.2 | 91.2 | 89.2 |
|  | 81 | Humidity | 76.5 | 85.3 | 88.2 | 83.3 |
|  | 82 | Field of view | 85.3 | 82.4 | 76.5 | 81.4 |
|  | 83 | Light intensity | 79.4 | 85.3 | 88.2 | 84.3 |
|  | 84 | Light source | 61.8 | 79.4 | 67.6^^^ | 70.6 |
|  | 85 | Noise levels | 94.1 | 79.4 | 58.8 | 77.4 |
|  | 86 | Cage position | 85.3 | 79.4 | 88.2 | 84.3 |
|  | 87 | Vibration | 67.6^^^ | 50.0 | 41.2 | 45.6 |
|  | 88 | Social density | 82.4 | 88.2 | 76.5 | 82.4 |
|  | 89 | Social stability | 97.1 | 64.7 | 55.9 | 72.6 |
|  | 90 | Spatial density | 67.6^^^ | 79.4 | 52.9 | 66.2 |
|  | 91 | Temperature of room | 85.3 | 94.1 | 97.1 | 92.2 |
|  | 92 | Ventilation | 94.1 | 94.1 | 94.1 | 94.1 |
|  | 93 | Vertical space | 85.3 | 85.3 | 79.4 | 83.3 |
|  | 94 | Visual barrier, between caging | 82.4 | 91.2 | 97.1 | 90.2 |
|  | 95 | Visual barrier, within caging | 82.4 | 88.2 | 82.4 | 84.3 |
|  | 96 | Behavioural management program | 100.0 | 88.2 | 94.1 | 94.1 |
|  | 97 | Canine blunting | 5.9^*^ | 17.6^*^ | 32.4 | 18.6 |
|  | 98 | Animal caregiver observations | 97.1 | 76.5 | 91.2 | 88.3 |
|  | 99 | Disease surveillance | 100.0 | 85.3 | 91.2 | 92.2 |
|  | 100 | Chair restraint frequency | 85.3 | 76.5 | 79.4 | 80.4 |
|  | 101 | Hand-catching frequency | 61.8 | 52.9 | 61.8 | 58.8 |
|  | 102 | Health monitoring | 100.0 | 94.1 | 100.0 | 98.0 |
|  | 103 | Humane euthanasia program | 100.0 | 94.2 | 100.0 | 98.1 |
|  | 104 | Inoculations, lifetime | 79.4 | 82.4 | 88.2 | 83.3 |
|  | 105 | Number of meals, daily | 76.5 | 79.4 | 97.1 | 84.3 |
| Environment-based | 106 | Timing of meals, daily | 73.5 | 73.5 | 91.2 | 79.4 |
|  | 107 | Moves, lifetime | 91.2 | 76.5 | 73.5 | 80.4 |
|  | 108 | Sedations, lifetime | 91.2 | 82.4 | 79.4 | 84.3 |
|  | 109 | Surgeries, lifetime | 97.1 | 85.3 | 94.1 | 92.2 |
|  | 110 | Experiments, lifetime | 73.5 | 67.6^^^ | 85.3 | 75.5 |
|  | 111 | Vet med procedures, lifetime | 82.4 | 76.5 | 79.4 | 79.4 |
|  | 112 | Staff training | 97.1 | 70.6 | 88.2 | 85.3 |
|  | 113 | Quality of life assessments | 85.3 | 44.1 | 73.5 | 67.6 |
|  | 114 | Rearing history | 100.0 | 85.3 | 76.5 | 87.3 |
|  | 115 | Weaning age | 85.3 | 76.5 | 76.5 | 79.4 |

Grey cell indicates agreement at a level of ≥ 70% that indicator is valid, reliable, or feasible. White cell without ^, *, or ** indicates agreement at a level of < 70% that indicator is valid, reliable, or feasible.

^^^Indicates approaching agreement at a level of 65-69.99% agreement.

^*^Indicates agreement at a level of ≥ 70% agreement that indicator is *not* valid, reliable, or feasible.

**Indicates agreement at a level of ≥ 70% agreement that indicator is *not* valid, reliable, and feasible.

**Supplementary Figure S5**. Example of round one survey.
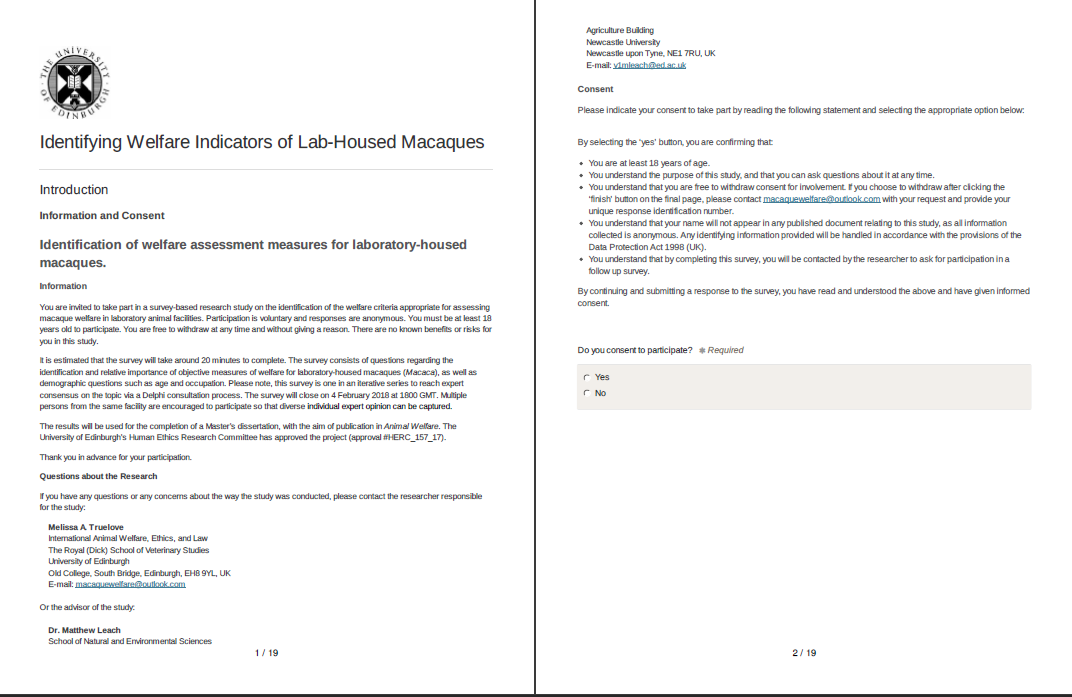

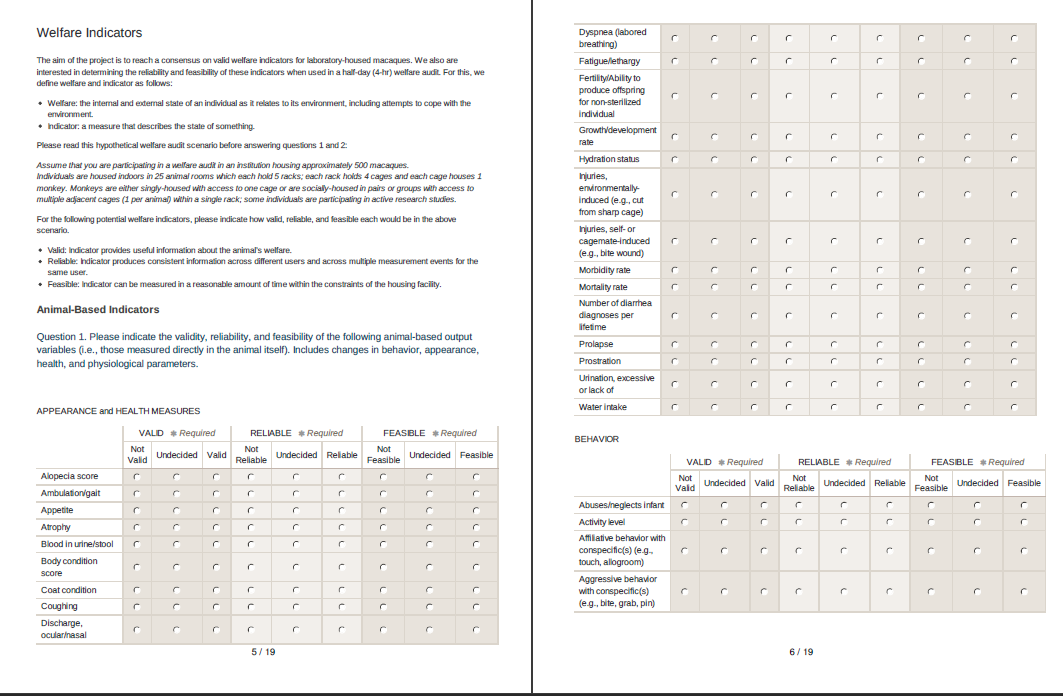

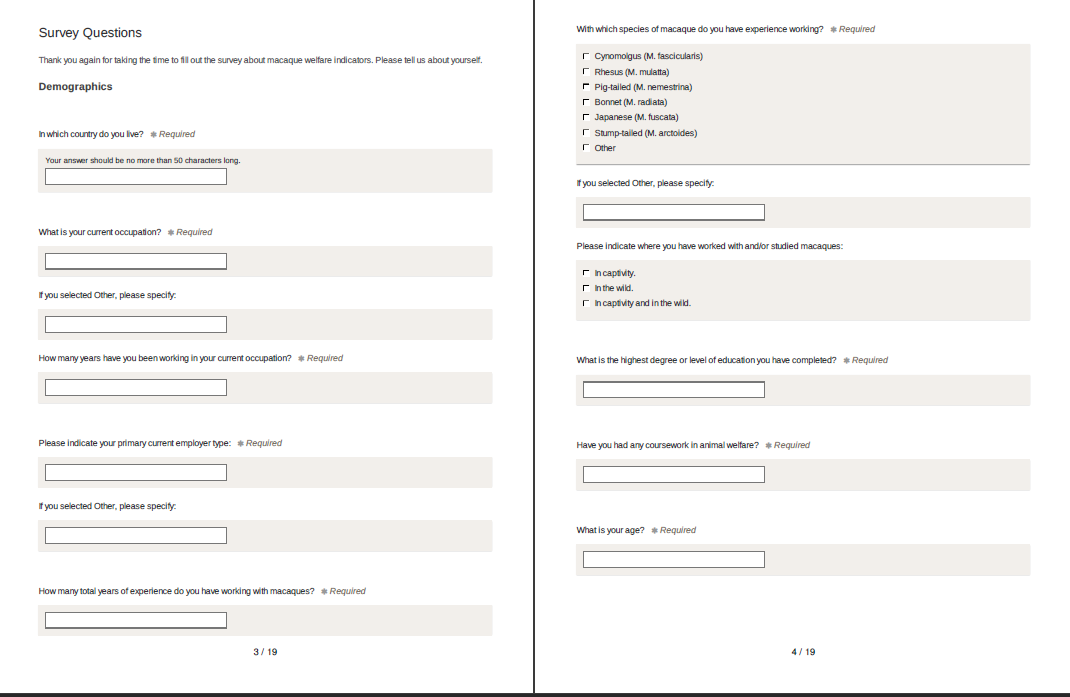


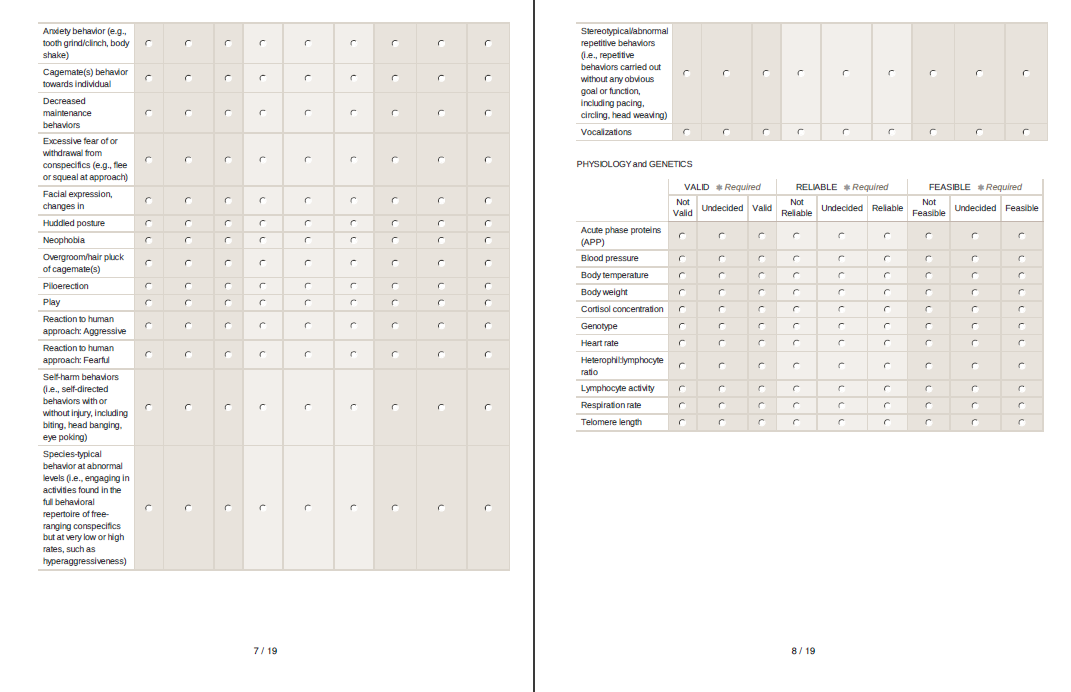


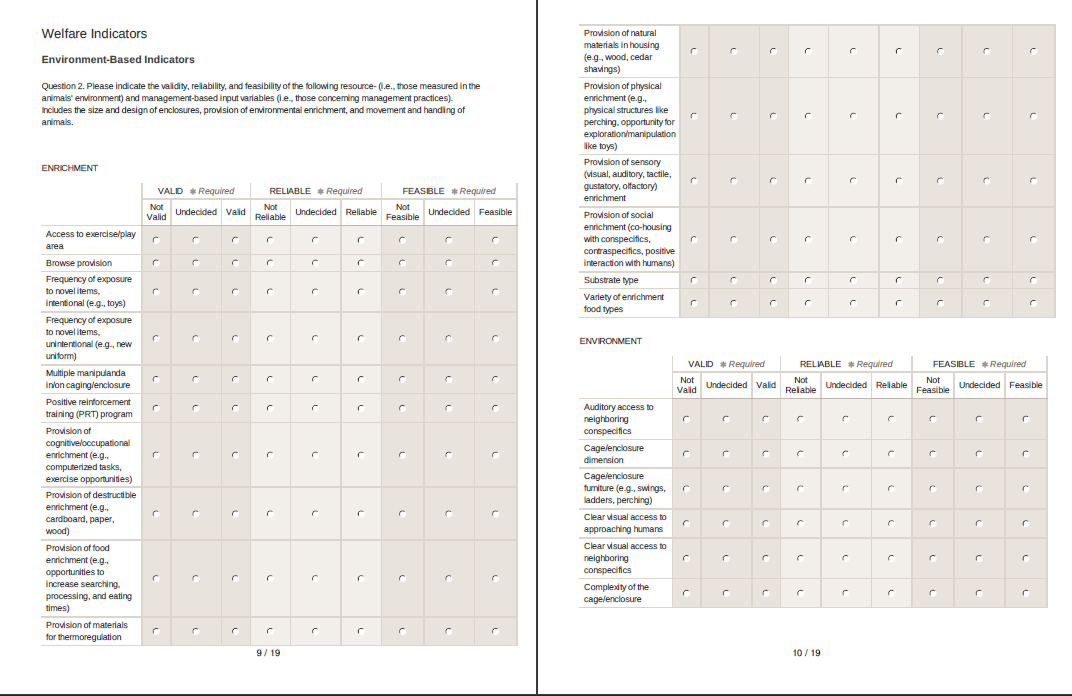


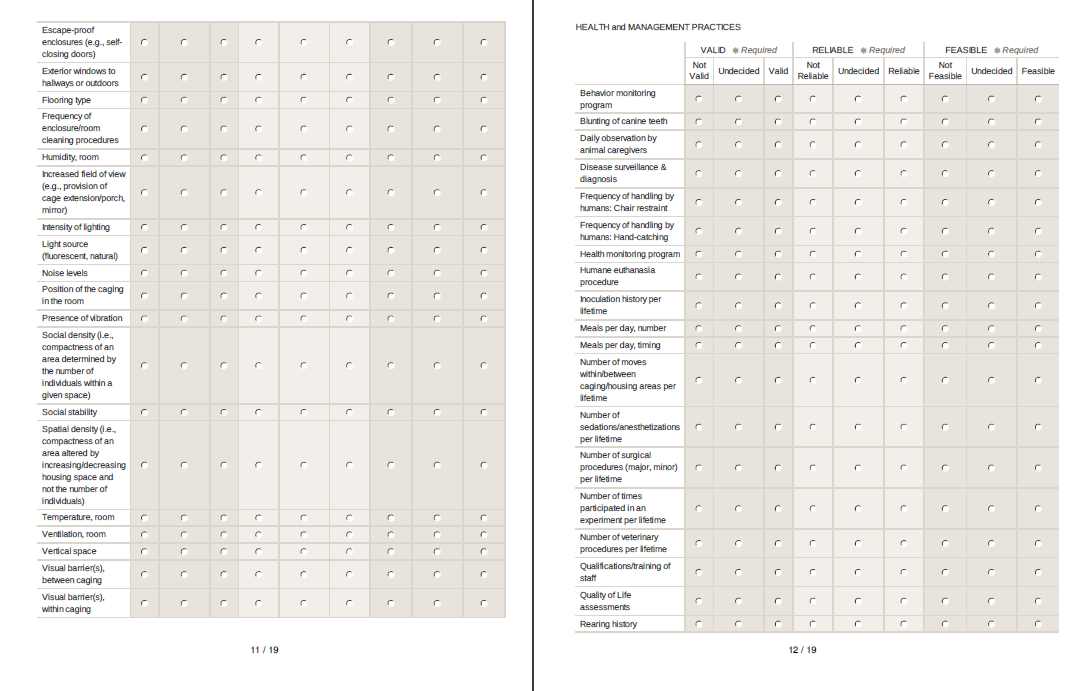


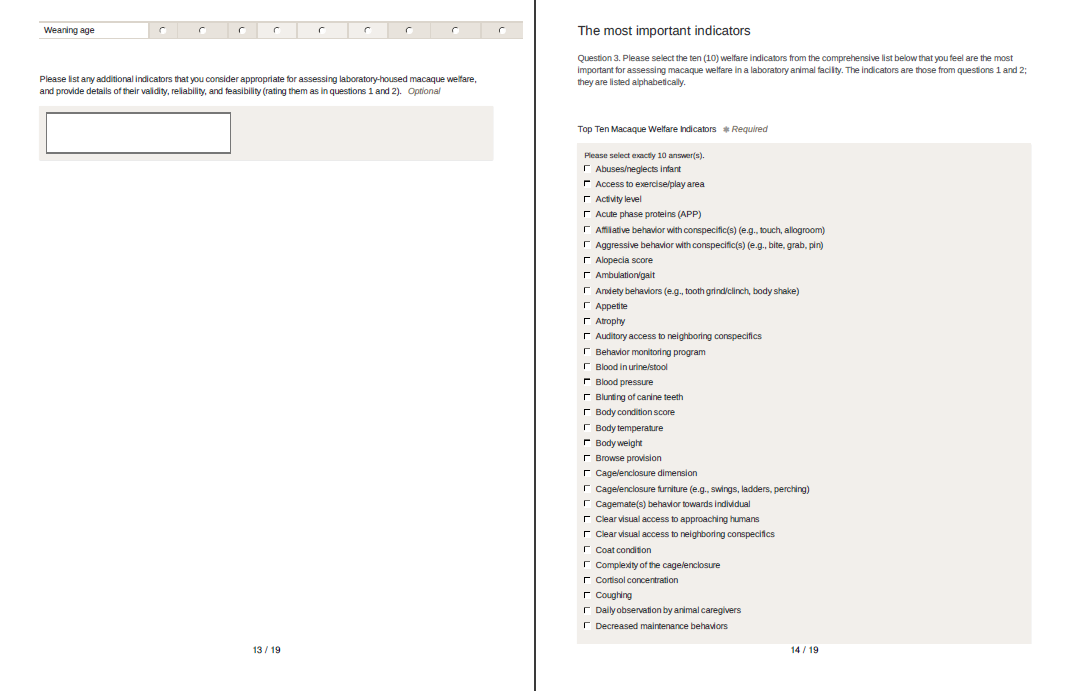


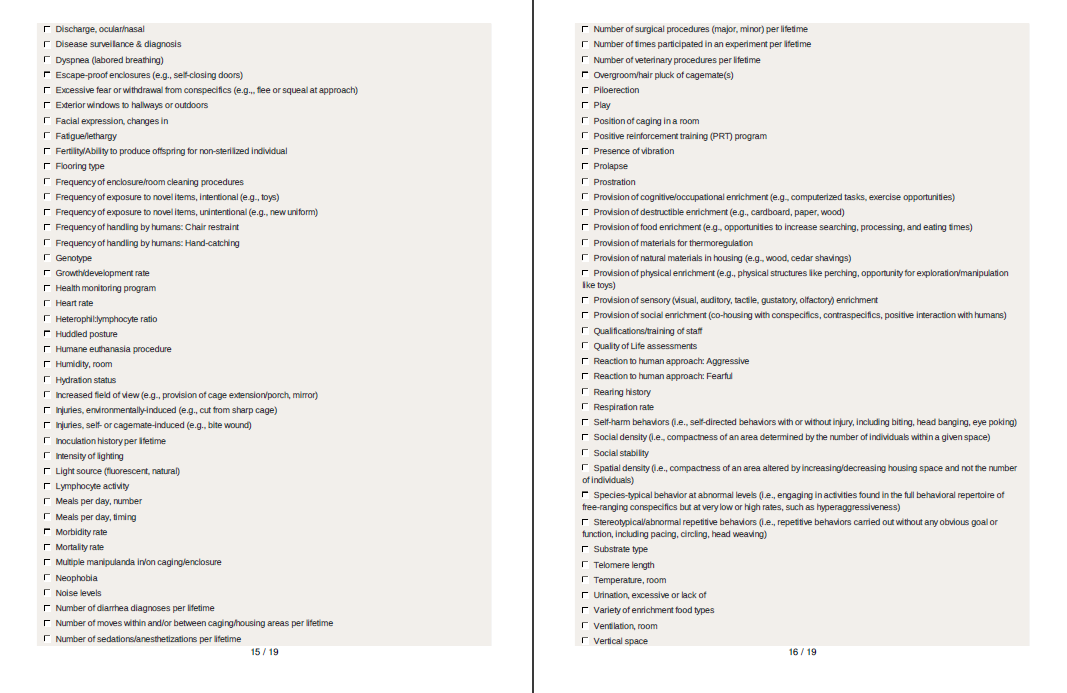


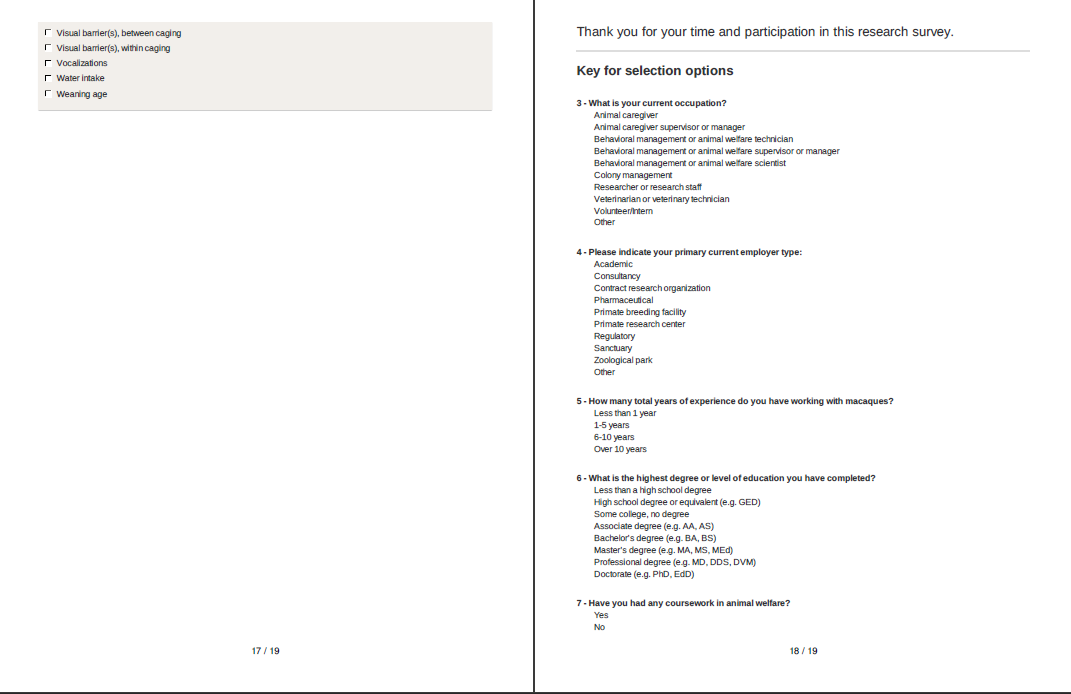


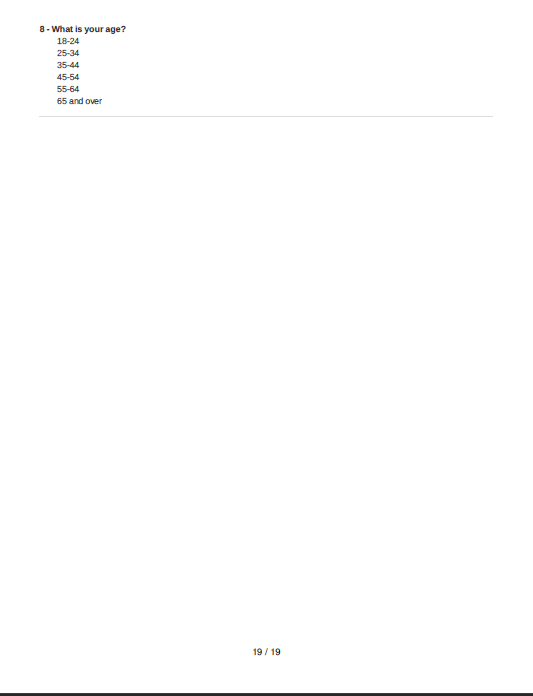


**Supplementary Figure S6.** Sample of portion of round two survey.

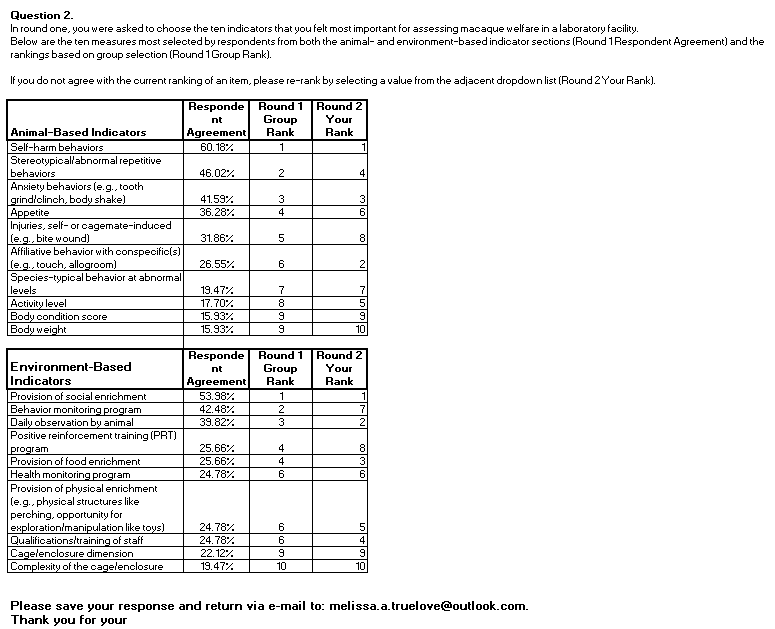


**Supplementary Figure S7**. Generalized linear mixed model (GLMM) descriptions.

| **Model #** | **Fixed effects** | **Random effect** |
| --- | --- | --- |
| 1 | Round, indicator, user ID | User ID |
| 2 | Round, indicator type (animal, environment), response type (validity, reliability, feasibility) | User ID |
